# Supplementary material for: An open-source framework for large-scale, flexible evaluation of biomedical text mining systems
Source: J Biomed Discov Collab. 2008 Jan 29;3:1. doi: 10.1186/1747-5333-3-1 (PMC2276192; doi:10.1186/1747-5333-3-1)
Supplement: Additional file 1 — Evaluation platform source code. The additional file contains the software evaluation framework discussed in this paper. The most current release can be downloaded from SourceForge.net [1]. See the accompanying README file for instructions on installation and use. Also included in the distribution is a collection of UIMA wrappers for some commonly used BioNLP tools and annotated corpora. [file 1747-5333-3-1-S1.zip › bionlp_uima_repository-1.0.1/docs/api/src-html/edu/uchsc/ccp/util/nlp/stats/NLPStats.html]

```
001    package edu.uchsc.ccp.util.nlp.stats;
002    
003    import java.util.Arrays;
004    import java.util.HashSet;
005    import java.util.Set;
006    
007    /**
008     * This class implements some commonly used statistics in the field of NLP, namely precision, recall, F-measure, and the Dice coefficient.
009     * <p>
010     * For a give count of true positives (TP), false positives (FP), and false negatives (FN), precision (P), recall (R), and F-measure (F) are computed
011     * as:
012     * <p>
013     * P = TP/(TP+FP)
014     * <p>
015     * R = TP/(TP+FN)
016     * <p>
017     * F = 2PR/(P+R)
018     * 
019     * @author William A Baumgartner, Jr.
020     * 
021     */
022    public class NLPStats {
023    
024        /**
025         * Given counts of TPs and FPs, compute the precision
026         * 
027         * @param tp
028         *            the number of true positives
029         * @param fp
030         *            the number of false positives
031         * @return TP/(TP+FP)
032         */
033        public static double computePrecision(int tp, int fp) {
034            return ((double) tp) / ((double) (tp + fp));
035        }
036    
037        /**
038         * Given counts of TPs and FNs, compute the recall
039         * 
040         * @param tp
041         *            the number of true positives
042         * @param fn
043         *            the number of false positives
044         * @return TP/(TP+FN)
045         */
046        public static double computeRecall(int tp, int fn) {
047            return ((double) tp) / ((double) (tp + fn));
048        }
049    
050        /**
051         * Given precision and recall, compute the F-measure
052         * 
053         * @param precision
054         * @param recall
055         * @return 2PR/(P+R)
056         */
057        public static double computeFMeasure(double precision, double recall) {
058            return 2.0 * precision * recall / (precision + recall);
059        }
060    
061        /**
062         * Given counts of TPs, FPs, and FNs, compute the F-measure
063         * 
064         * @param tp
065         * @param fp
066         * @param fn
067         * @return 2PR/(P+R)
068         */
069        public static double computeFMeasure(int tp, int fp, int fn) {
070            return 2.0 * computePrecision(tp, fp) * computeRecall(tp, fn) / (computePrecision(tp, fp) + computeRecall(tp, fn));
071        }
072    
073        /**
074         * Computes the Dice Coefficient as described in Hersh et al. TREC Genomics Track Overview, 2003.
075         * <p>
076         * For two <code>Strings</code> A and B, define X as the number of words in A, Y as the number of words in B, and Z as the number of words
077         * occurring in both A and B. The Dice coefficient is calculated as Dice(A,B) = (2*Z)/(X+Y)
078         * 
079         * @param aTokens
080         *            a <code>Set</code> of tokens from <code>String</code> A
081         * @param bTokens
082         *            a <code>Set</code> of tokens from <code>String</code> B
083         * @return the Dice Coefficient for the two sets of tokens
084         */
085        public static double computeClassicDice(Set<String> aTokens, Set<String> bTokens) {
086            int numWordsInA = aTokens.size();
087            int numWordsInB = bTokens.size();
088            aTokens.retainAll(bTokens);
089            int numWordsThatCoOccur = aTokens.size();
090    
091            double diceCoefficient = ((double) (2 * numWordsThatCoOccur)) / ((double) (numWordsInA + numWordsInB));
092            return diceCoefficient;
093        }
094    
095        /**
096         * Computes the Dice Coefficient as described in Hersh et al. TREC Genomics Track Overview, 2003.
097         * <p>
098         * For two <code>Strings</code> A and B, define X as the number of words in A, Y as the number of words in B, and Z as the number of words
099         * occurring in both A and B. The Dice coefficient is calculated as Dice(A,B) = (2*Z)/(X+Y)
100         * <p>
101         * This method tokenizes by splitting the input Strings at spaces.
102         * 
103         * @param aString
104         *            a <code>String</code> to compare
105         * @param bString
106         *            another <code>String</code> to compare
107         * @return the Dice Coefficient for the two input Strings
108         */
109        public static double computeClassicDice(String aString, String bString) {
110            Set<String> aTokens = new HashSet<String>(Arrays.asList(aString.split(" ")));
111            Set<String> bTokens = new HashSet<String>(Arrays.asList(bString.split(" ")));
112            return computeClassicDice(aTokens, bTokens);
113    
114        }
115    
116    }
```
